# Supplementary material for: Optogenetic activation of Plexin-B1 reveals contact repulsion between osteoclasts and osteoblasts
Source: Nat Commun. 2017 Jun 21;8:15831. doi: 10.1038/ncomms15831 (PMC5482063; doi:10.1038/ncomms15831)
Supplement: Supplementary Information [file ncomms15831-s21.pdf]

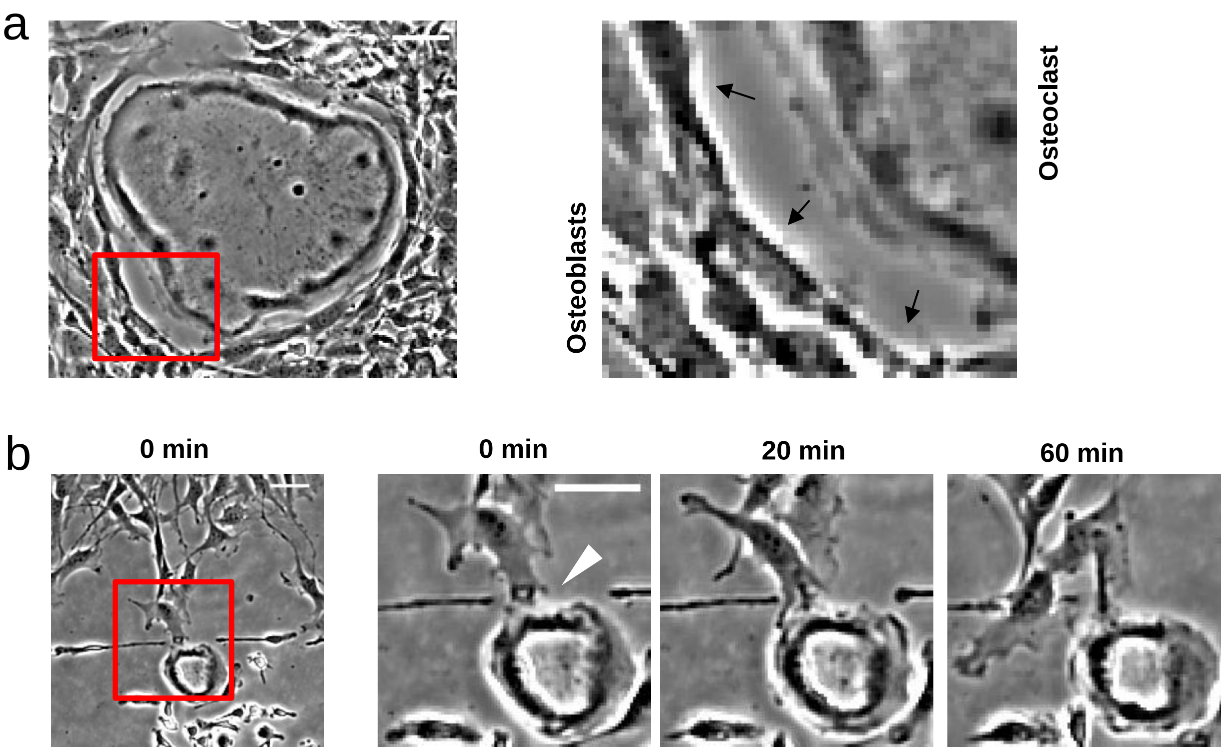

Supplementary Figure 1. (a) Phase contrast images showing separation between osteoclasts and osteoblasts in a co-culture. The inset (106 μm X 92 μm) is magnified on the right panel to show the gap between the osteoclast and the osteoblasts and the lack of protrusions in osteoblasts. The arrows point towards the osteoblasts. **(b)** Phase contrast images showing contact between an osteoblast and an osteoclast. The inset is magnified to show changes in cell morphology and migration between time of contact and 20 and 40 minutes after contact. White triangle points to the site of cell-cell contact. Scale bar, 50 μm.

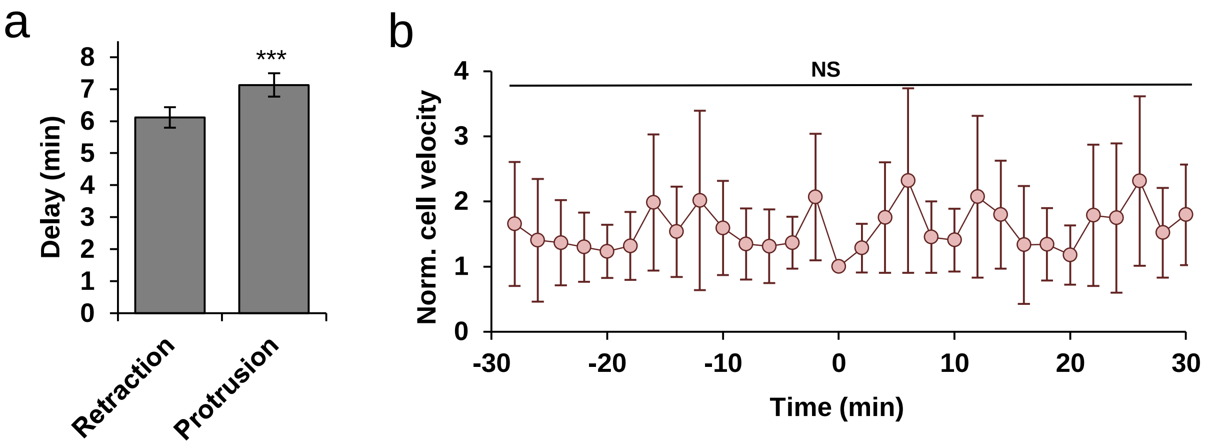

Supplementary Figure 2. (a) Delay in initiation of retraction at the site of contact and initiation of new distal protrusions in WT MC3T3-E1 cells upon contact with osteoclasts. n=85 cells, mean ± s.e.m. are shown. **(b)** Velocities of MC3T3-E1 cells coming in contact with osteoclasts, normalized to the velocity right before contact at time 0. n=29 cells, mean ± s.e.m. are shown. *** $p<0.001$, * $p<0.05$, NS, not significant, Student's t-test.

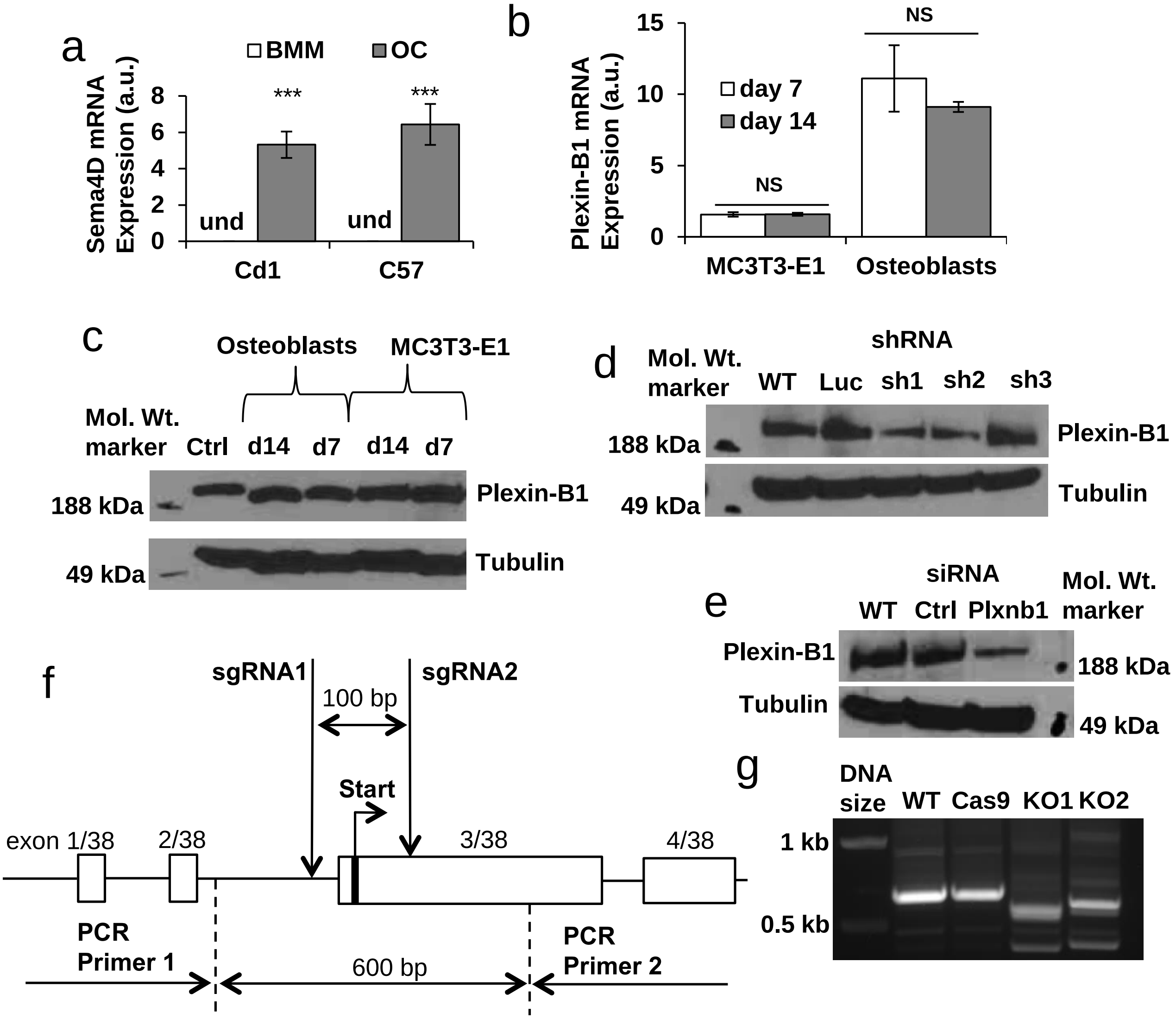

Supplementary Figure 3. (a) Comparison of Sema4D mRNA expression in pre-osteoclasts (BMMs treated with M-CSF) and osteoclasts (BMMs treated with M-CSF and RANKL) from Cd1 and C57BL/6 mice. Osteoclastogenesis was confirmed by visual inspection of formation of multinucleated cells on fourth day of RANKL treatment. und, undetectable. **(b)** Comparison of Plexin-B1 mRNA expression in MC3T3-E1 cells and primary calvarial osteoblasts from C57BL/6 mice. mRNA were extracted after 7 and 14 days of phosphoascorbate mediated differentiation. **(c)** Comparison of Plexin-B1 expression in MC3T3-E1 and pimary calvarial osteoblasts from C57BL/6 mice after 7 and 14 days of maintenance in differentiating media. COS-7 cells overexpressing Plexin-B1 were used for positive control. **(d)** Comparison of Plexin-B1 expression in WT MC3T3-E1 cells and MC3T3-E1 cells stably expressing shRNA against Luciferase (Luc) or Plexin-B1 (sh1, sh2 and sh3). **(e)** Comparison of Plexin-B1 expression in WT MC3T3-E1 cells and cells transiently transfected with a non-targeting siRNA pool (Ctrl) or Plexin-B1 siRNA pool (PlxnB1). **(f)** Cartoon representation of the sgRNA design strategy for CRISPR-Cas9 mediated Plexin-N1 knock out (see main text). **(g)** Comparison of PCR product using primer 1 and 2 on genomic DNA in WT, Cas9, KO1 and KO2 MC3T3-E1 cells to probe for deletion targeted by sgRNA1 and sgRNA2. For a & b means ± s.e.m. are shown, n=3 wells of cells. *** $p<0.001$, * $p<0.05$, NS, not significant, One-way ANOVA. For c, d and e tubulin was used as a loading control.

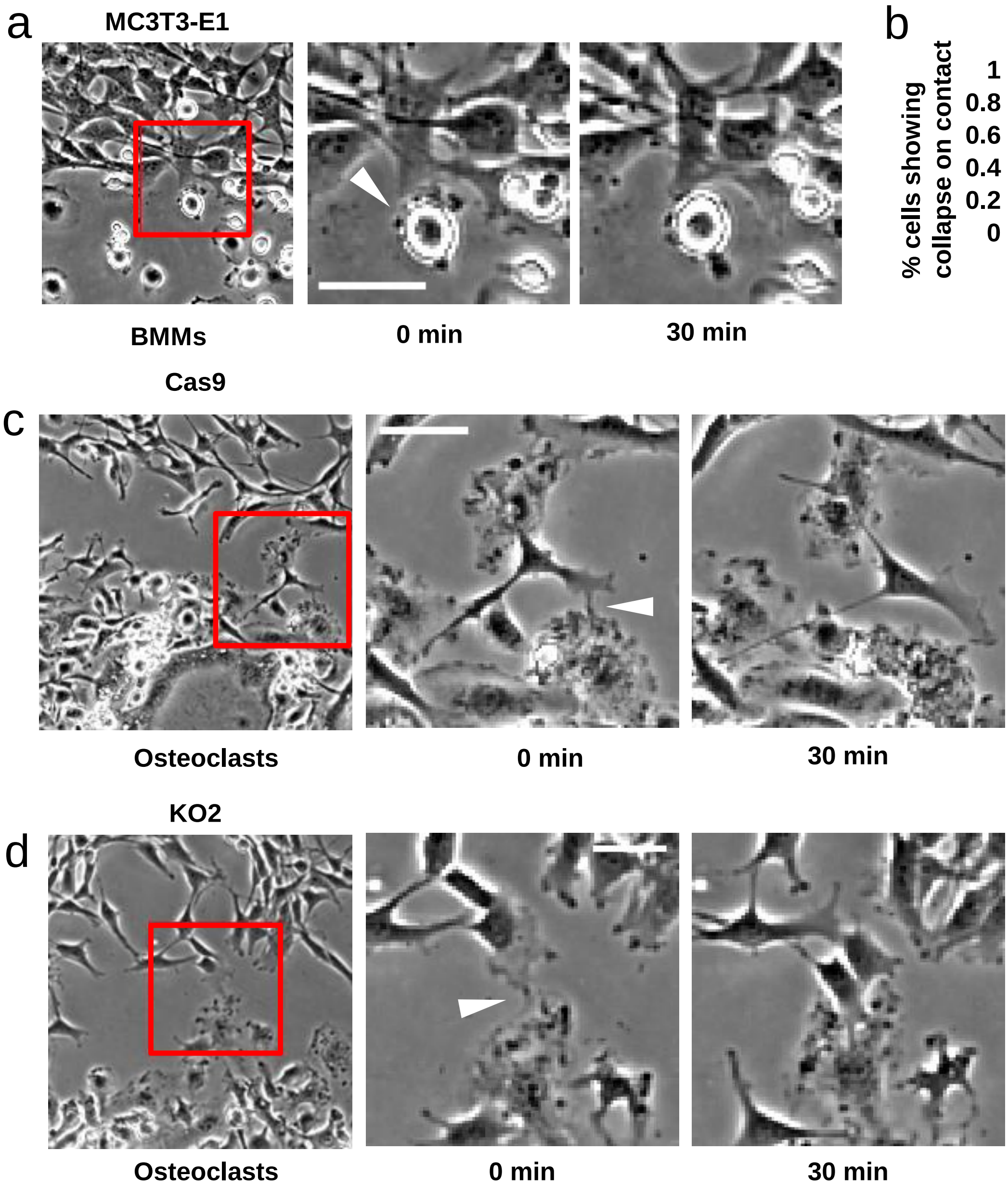

Supplementary Figure 4. (a) Phase contrast images showing contact between a MC3T3-E1 cells and a BMM. **(b)** Percentage of MC3T3-E1 cells that show collapse of protrusions within 30 minutes after contact with a BMM or an osteoclast. n = 198-301. ***, p<0.001, Student's t-test. **(c), (d)** Phase contrast images showing contact between MC3T3-E1 Cas9 **(c)** or MC3T3-E1 KO2 **(d)** cell and an osteoclast. The inset is magnified to show changes in cell morphology and migration between time of contact and 30 minutes after contact. Scale bar, 50 µm. White triangles point to the site of cell-cell contact.

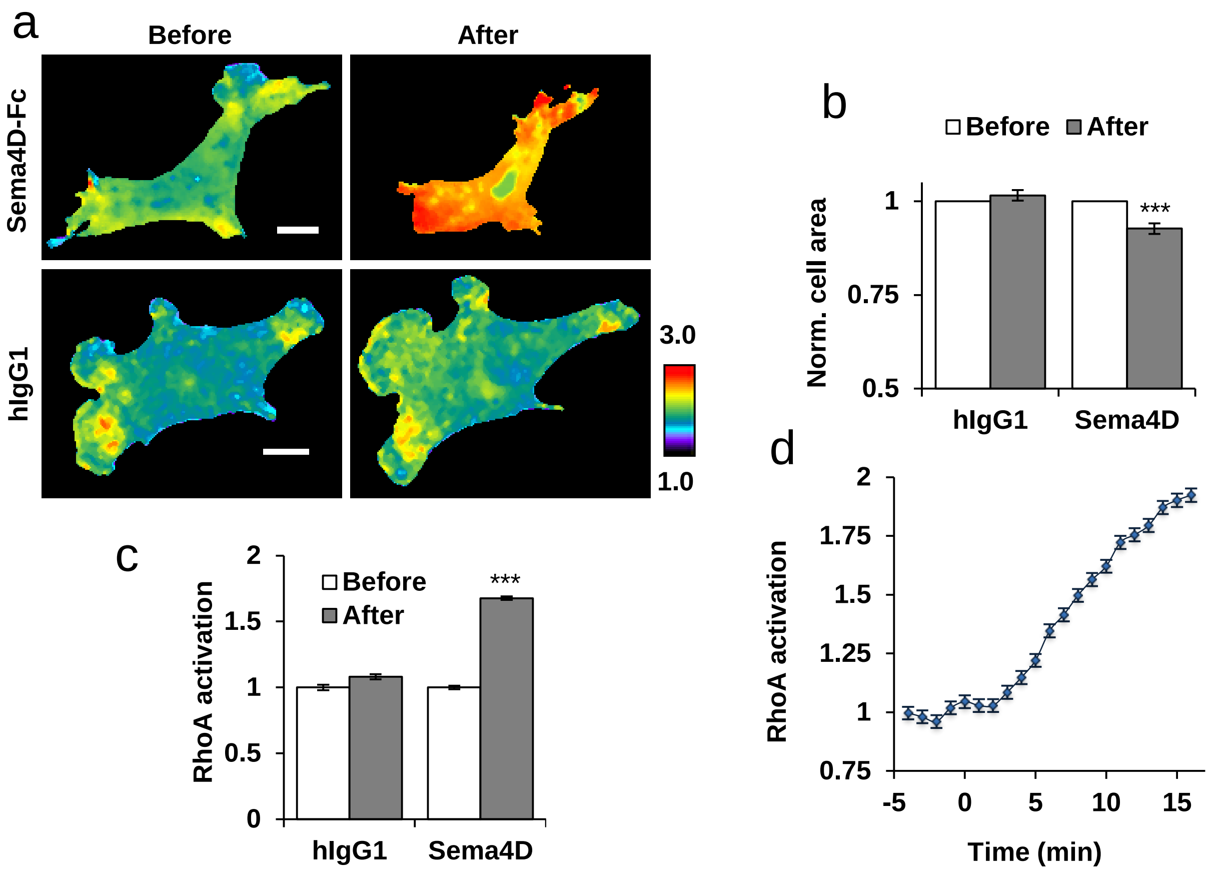

Supplementary Figure 5. (a) Ratiometric images showing changes in morphology and RhoA activation in MC3T3-E1 cells expressing DORA-RhoA on treatment with 200 nM Sema4D-Fc or hIgG1. Scale bar, 10 µm. **(b)** Changes in cell area of MC3T3-E1 cells treated with 200 nM Sema4D-Fc or 200 nM hIgG1, normalized to cell area before treatment. n ≥ 11 cells. **(c)** Changes in RhoA activity of MC3T3-E1 cells treated with 200 nM Sema4D-Fc or 200 nM hIgG1, normalized to activities before treatment. n ≥ 11 cells. **(d)** Temporal changes in RhoA activity in MC3T3-E1 cells treated with 200 nM Sema4D-Fc. n=17 cells. For b,c and d means ± s.e.m. are shown. *** p<0.001, * p<0.05, NS, not significant, Student's t-test.

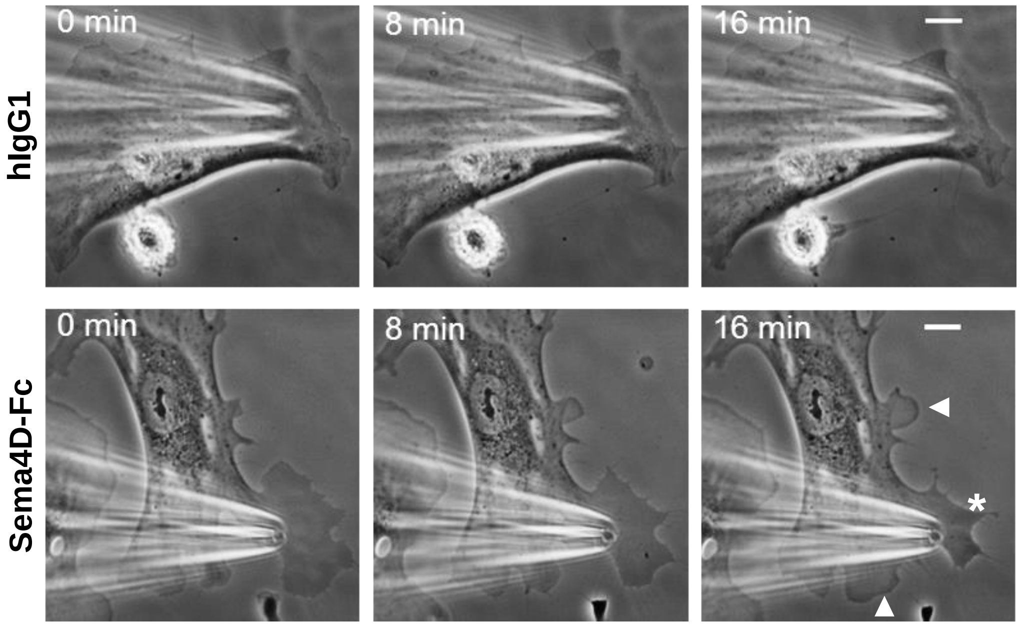

Supplementary Figure 6. Representative DIC images showing changes in morphology in murine calvarial osteoblasts from C57BL/6 mice upon localized Sema4D-Fc or hIgG1(control) perfusion. Calvarial osteoblasts were differentiated for 7 days before imaging. 1 μM Sema4D-Fc or hIgG1 was perfused at protrusions using positive pressure through a micropipette. Local perfusion of Sema4D-Fc induced local retractions and distal protrusions in osteoblasts (4 out of 5). No morphological changes observed in response to local perfusion of hIgG1 (3 out of 3). Scale bar, 10 μm. Asterisk, induced retraction. Arrowhead, induced protrusion.

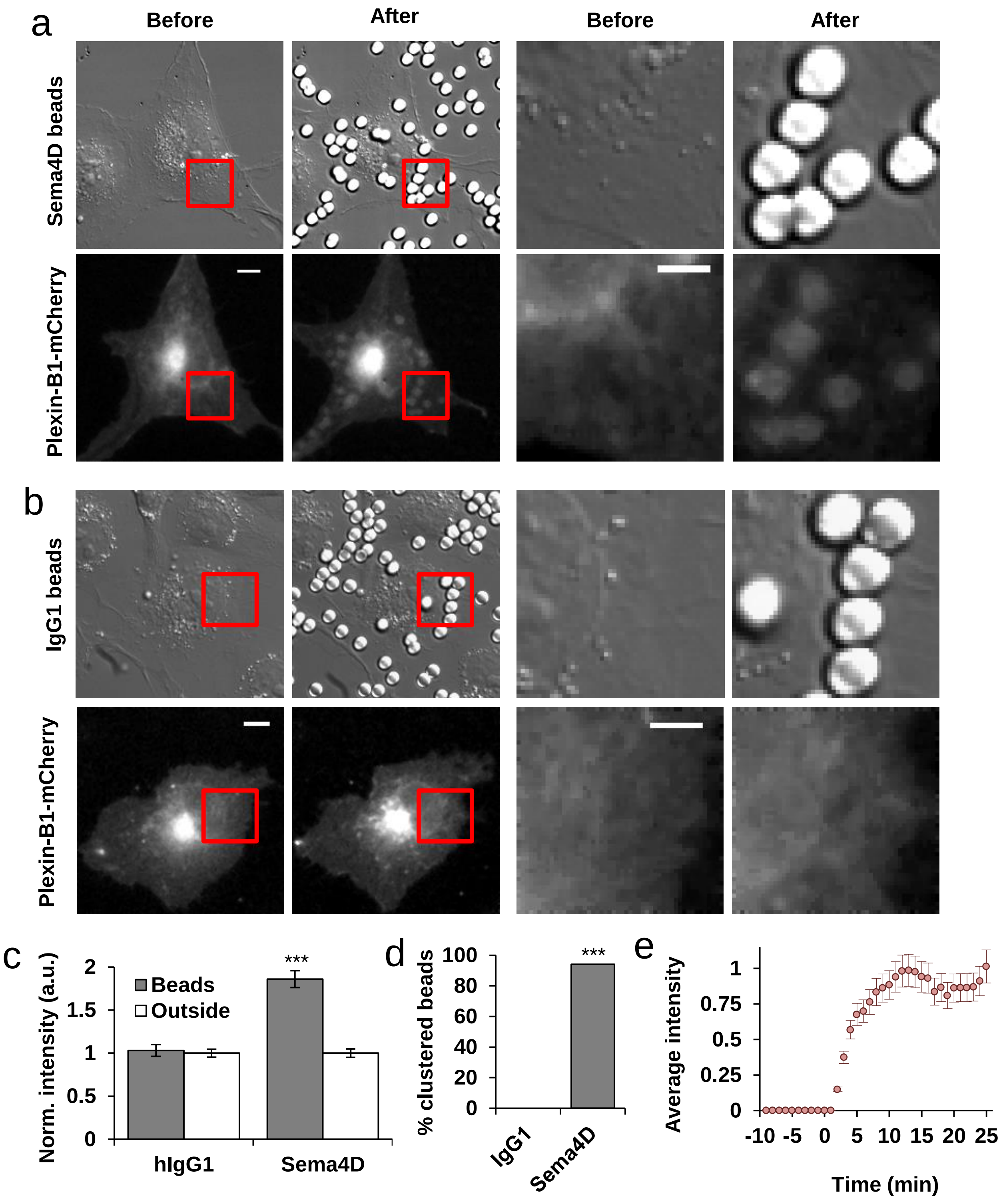

Supplementary Figure 7. DIC and wide-field fluorescent images showing changes in Plexin-B1-mCherry distribution in COS-7 cells before and 10 minutes after contact with Sema4D-Fc **(a)** or hIgG1 **(b)** immobilized on silica beads. Scale bar, 10 μm. The insets are magnified to show changes in distributionPlexin-B1-mCherry around Sema4D-Fc beads or hIgG1 beads. Scale bar, 5 μm. **(c)** mCherry intensity in 5 μm circle around beads and beads free cell body after 10 minutes of contact with beads, normalized to intensity in beads free regions. n = 70-90 regions, mean ± s.e.m. are shown. **(d)** Percentage of beads that show clustering of mCherry around it. n = 230-250 beads. **(e)** Temporal changes in Plexin-B1-mCherry clustering around Sema4D-Fc beads. n= 5 cells, mean ± s.e.m. are shown. *** p<0.001, * p<0.05, NS, not significant, Student's t-test.

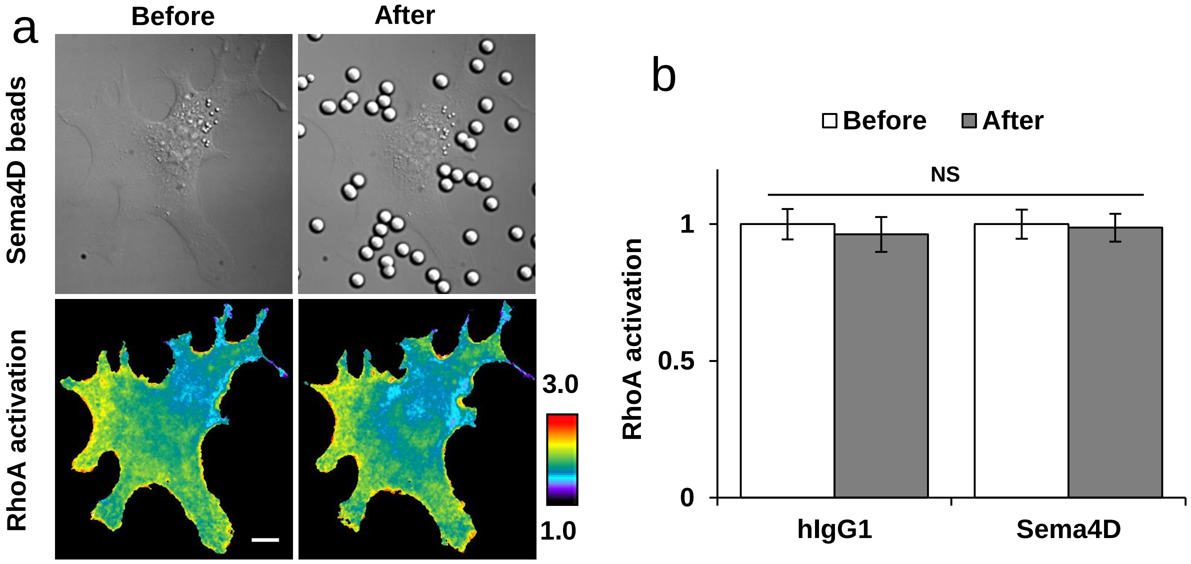

Supplementary Figure 8. (a) DIC and ratiometric images showing changes in morphology and RhoA activation in MC3T3-E1 cells expressing DORA-RhoA before and after contact with Sema4D-Fc beads. Scale bar, 10 μm. **(b)** Changes in RhoA activity of MC3T3-E1 cells after 10 minutes of contact with Sema4D-Fc of hIgG1 beads, normalized to activities before contact. n = 7-10 cells, means ± s.e.m. are shown. *** p<0.001, * p<0.05, NS, not significant, Student's t-test.

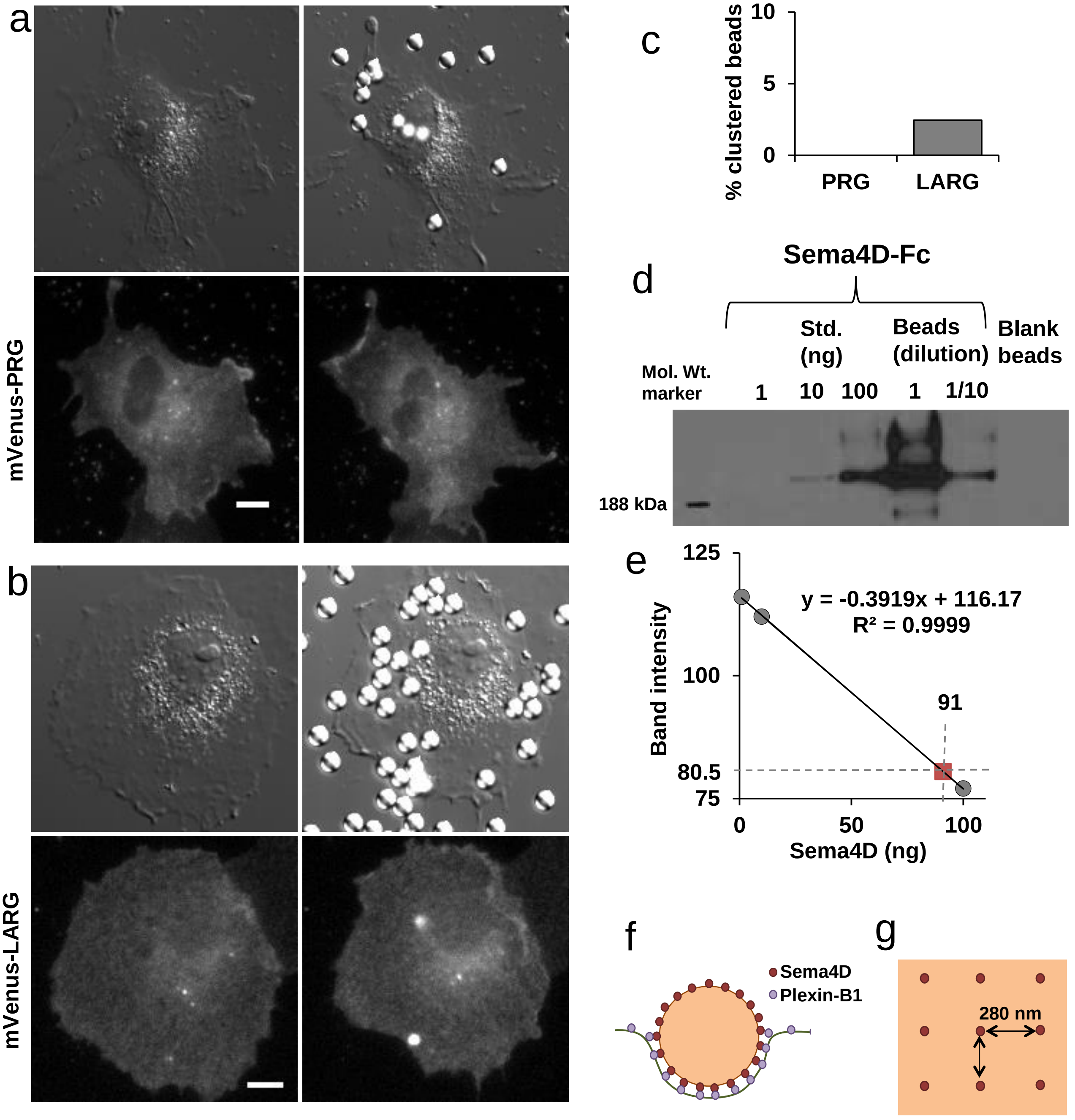

Supplementary Figure 9. DIC and wide-field fluorescent images showing changes in mVenus-PRG **(a)** or mVenus-LARG **(b)** distribution around Sema4D-Fc beads in COS-7 cells exogenously expressing Plexin-B1 with mVenus-PRG or mVenus-LARG. Scale bar, 10 µm. **(c)** Percentage of beads that show mVenus clustering around it. n = 90-125 beads. **(d)** Comparison of Sema4D-Fc bound to silica beads. **(e)** Analysis of the western blot in **(c)** to assess the amount of Sema4D-Fc bound to the silica beads. Gray circles indicate the standards. Red square corresponds to the intensity of the beads dilution 1/10. **(f)** Cartoon representation of hemisperic contact between Sema4D-Fc silica beads and cells and subsequent Plexin-B1 clustering around the beads. **(g)** Cartoon representation of the estimated distance between Sema4D-Fc molecules immobilized on a silica beads.

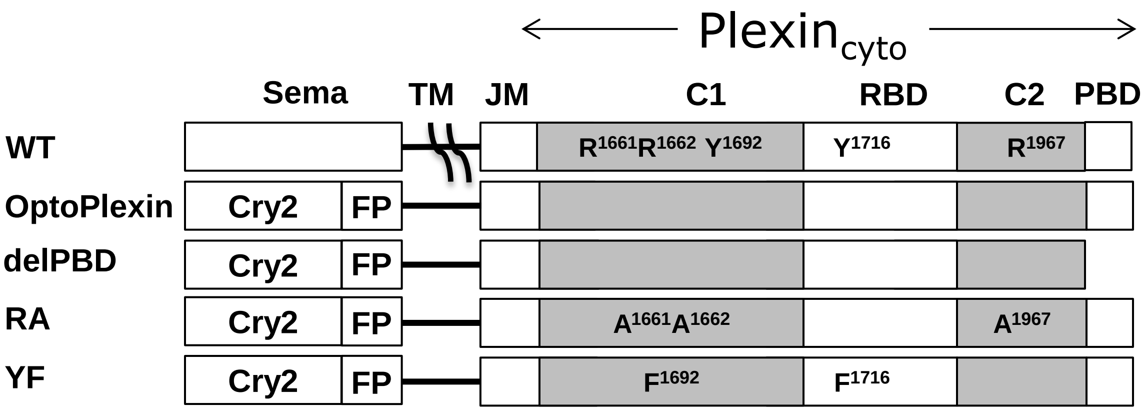

Supplementary Figure 10. A diagram depicting the domain organization of Plexin-B1, optoPlexin and its mutants. $\text{Plexin}_{\text{cyto}}$, intracellular domain of Plexin-B1. WT, wild type. Sema, Sema domain. TM, transmembrane region. JM, juxtamembrane region. C1 and C2, N and C-terminal halves of Plexin-B1 RasGAP domain. RBD, Rho GTPase binding domain that binds to Rac1 and Rnd1. PBD, PDZ binding domain. FP, fluorescent protein.

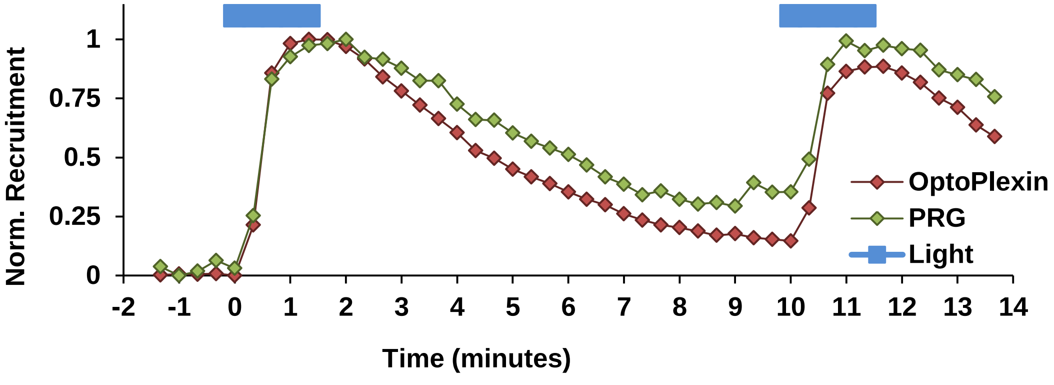

Supplementary Figure 11. Representative temporal changes in membrane recruitment and dissociation of mCherry-optoPlexin and mVenus-PRG in response to intermittent whole cell illumination in a COS-7 cell. Blue line, illumination at 440 nm.

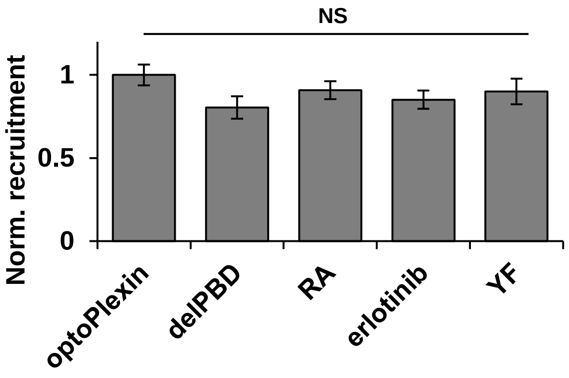

Supplementary Figure 12. Recruitment levels of optoPlexin, optoPlexin-delPBD, optoPlexin-RA, optoPlexin with 5 μM erlotinib and optoPlexin-YF on whole cell blue light illumination in COS-7 cells, normalized to recruitment level of optoPlexin. n = 15-19 cells, means ± s.e.m. are shown. *** $p<0.001$, * $p<0.05$, NS, not significant, Student's t-test.

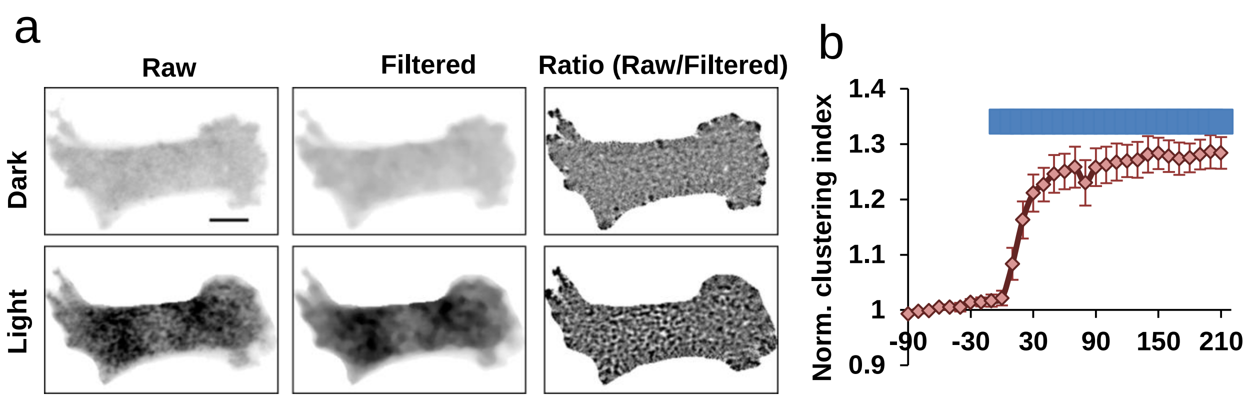

Supplementary Figure 13. (a) TIRF images showing changes in mCherry-optoPlexin clustering (left panels) in COS-7 cells before and after whole cell illumination. Images in right panel were passed through a 5X5 median filter to obtain 'smooth' images (middle panels). Changes in clustering upon illumination was visualized (right panels) by normalizing the raw images (left panels) to the filtered images (middle panels) to minimize effects of cell topology. Scale bar, 10 µm. **(b)** Temporal changes in optoPlexin clustering normalized to clustering levels prior to illumininination. Blue line, illumination. n=14 cells, mean ± s.e.m. are shown.

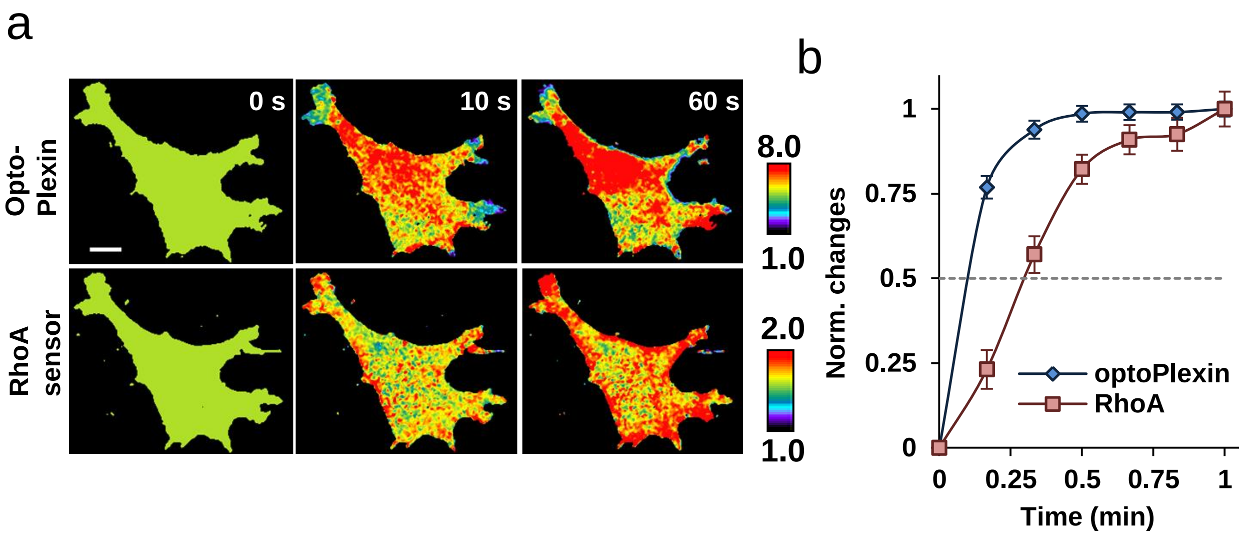

Supplementary Figure 14. (a) TIRF images showing changes in mCherry-optoPlexin and Dora-RhoA biosensor induction in COS-7 cells normalized to the first frame. Scale bar, 10 µm. **(b)** Temporal changes in optoPlexin stimulation and Dora-RhoA biosensor activity in COS-7 cells. Excitation wavelengths used for donor (CFP) and FRET images were sufficient for whole cell induction of optoPlexin. Mean ± s.e.m are shown, n=32 cells.

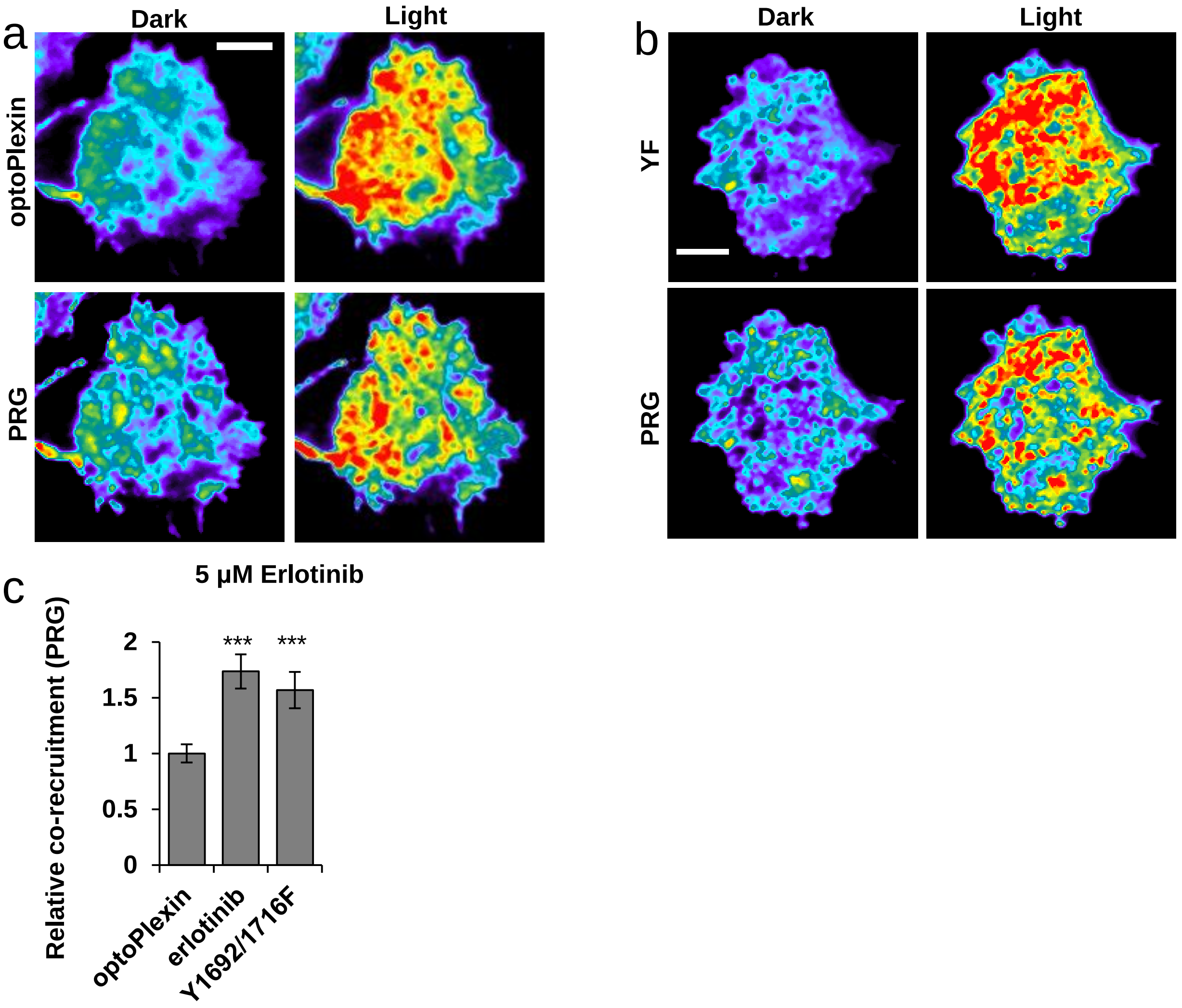

Supplementary Figure 15. TIRF images showing the membrane recruitment of mVenus-PRG and **(a)** mCherry-optoPlexin + 5 μM erlotinib or **(b)** mCherry-optoPlexin-YF after one minute of whole cell illumination in COS-7 cells. Scale bar, 10 μm. **(c)** Induction of mVenus-PRG relative to that of optoPlexin, optoPlexin + 5 μM erlotinib and optoPlexin-YF upon whole cell illumination in COS-7 cells, n = 15-19 cells, mean ± s.e.m. are shown. *** $p<0.001$, * $p<0.05$, NS, not significant, Student's t-test.

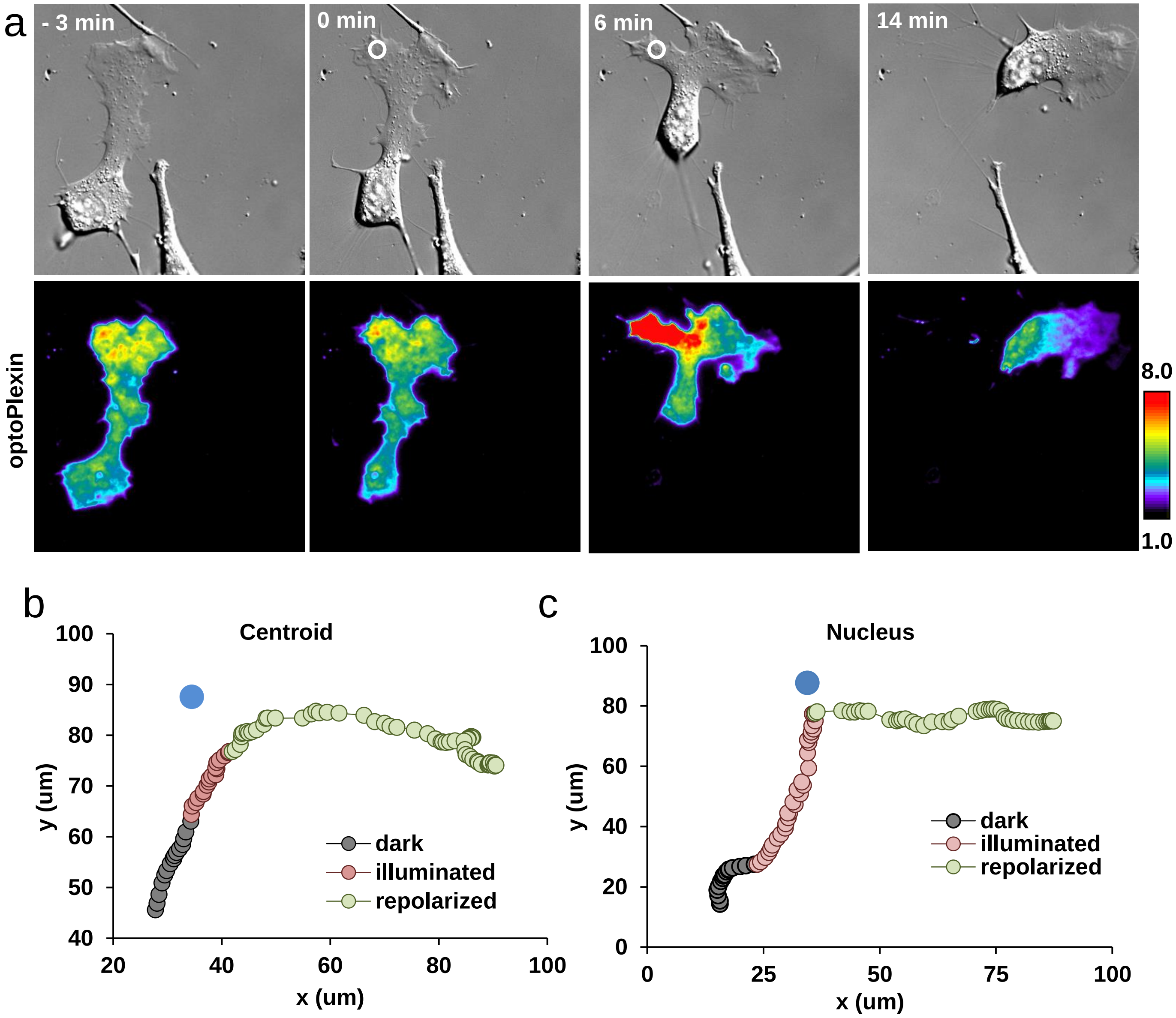

Supplementary Figure 16. (a) MC3T3-E1 cell expressing mCherry-optoPlexin was locally illuminated as indicated (white circle). The DIC and TIRF images were shown to illustrate the local membrane recruitment of mCherry-optoPlexin, and change in direction of migration. Scale bar, 10 μm. Asterisk, retraction. Arrowhead, induced protrusion. Changes in the direction of migration of the cell is illustrated by the changes in the centroid **(b)** and nucleus **(c)** of the cell. The change in direction of migration of the cenroid appears to be more gradual and precedes that of the nucleus. Blue circle, initial region of illumination.

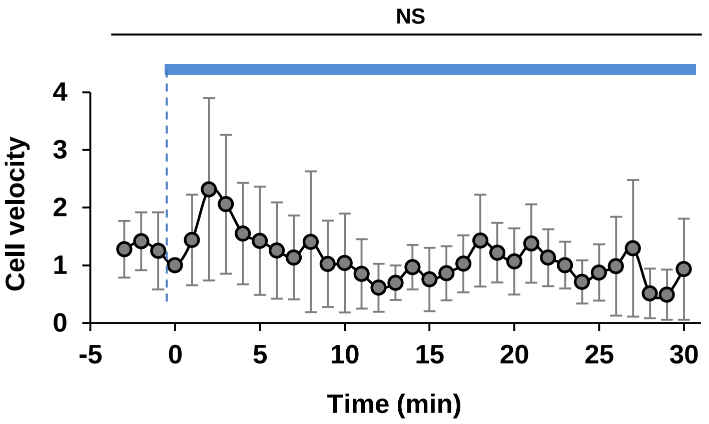

Supplementary Figure 17. Cell nucleus velocities at different times upon local activation of optoPlexin in MC3T3-E1 cells. Blue line, illumination at 440 nm. n=21 cells, mean ± s.e.m. are shown. *** p<0.001, * p<0.05, NS, not significant.

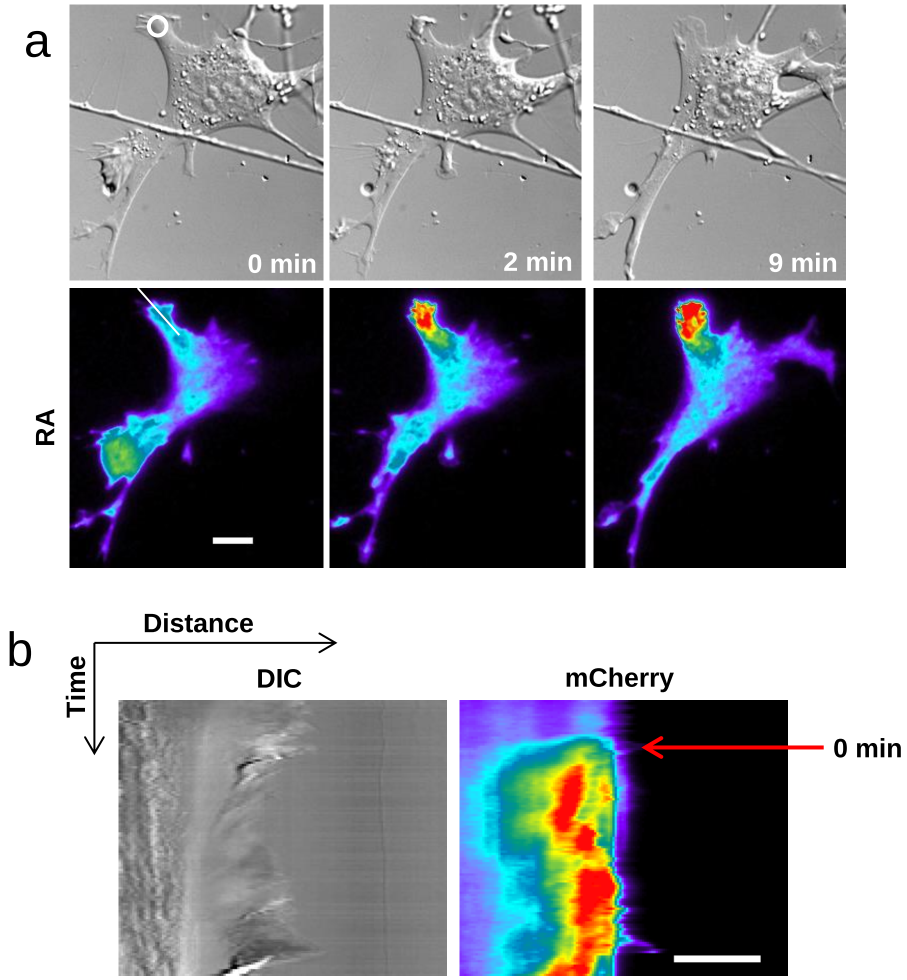

Supplementary Figure 18. (a) MC3T3-E1 cells expressing mCherry-optoPlexin-RA were locally illuminated as indicated (white circle). The DIC and TIRF images were shown to illustrate the morphological changes and local membrane recruitment. Scale bar, 10 µm. **(b)** Kymograph showing cell border progression upon local activation of optoPlexin-RA. Reference line for the kymograph is shown in white in **(a)**. Scale bar, 10 µm.

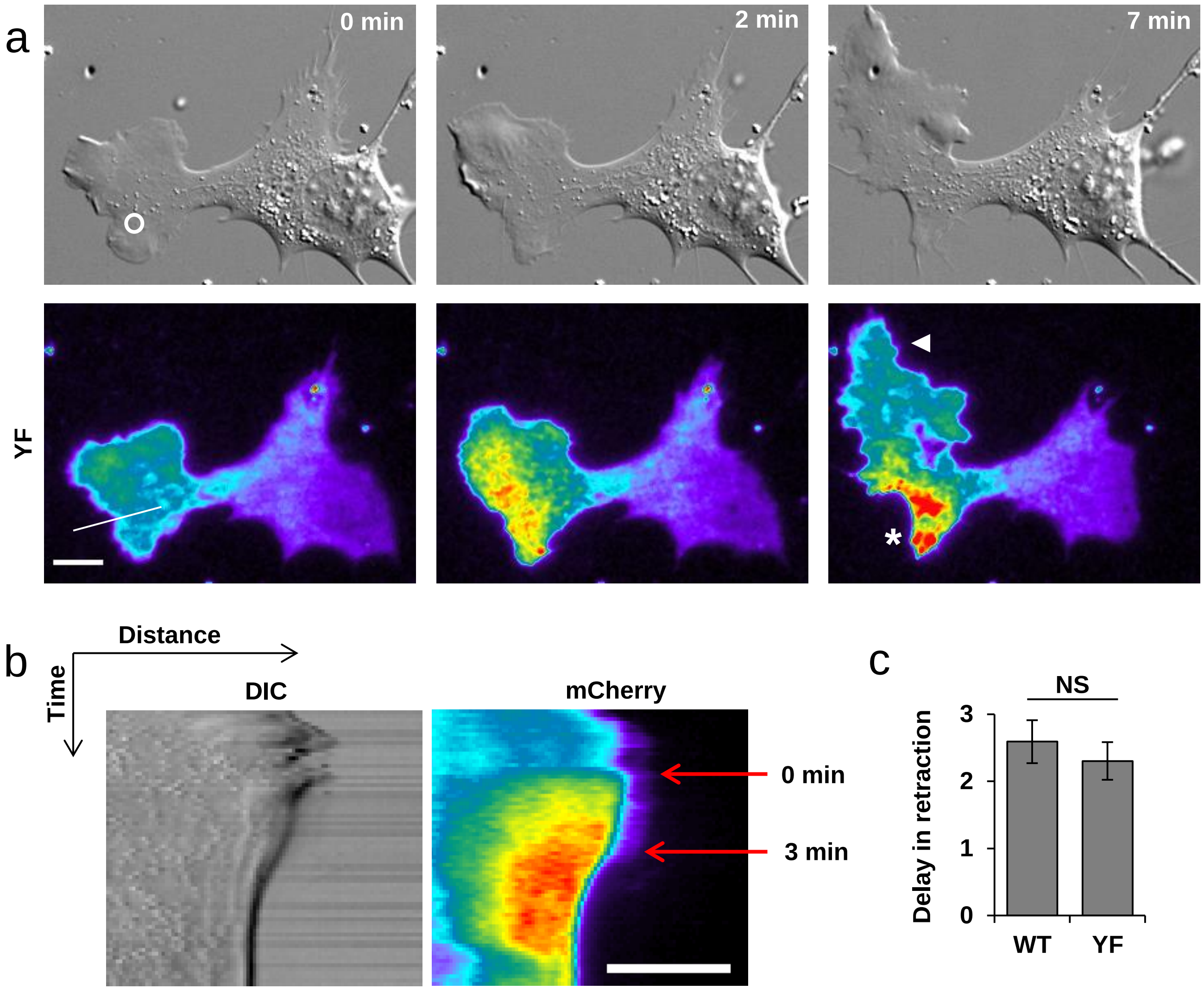

Supplementary Figure 19. (a) MC3T3-E1 cells expressing mCherry-optoPlexin-YF were locally illuminated as indicated (white circle). The DIC and TIRF images were shown to illustrate the morphological changes and local membrane recruitment. Scale bar, 10 µm. Asterisk, retraction. Arrowhead, induced protrusion. **(b)** Kymograph showing cell border progression upon local activation of optoPlexin-YF. Reference line for the kymograph is shown in white in **(a)**. Scale bar, 10 µm. **(c)** Comparison of delay in initiation of local retractions on local optoPlexin and optoPlexin-YF activation. n = 10-14 cells, means ± s.e.m. *** $p<0.001$, * $p<0.05$. NS, not significant, Student's t-test.

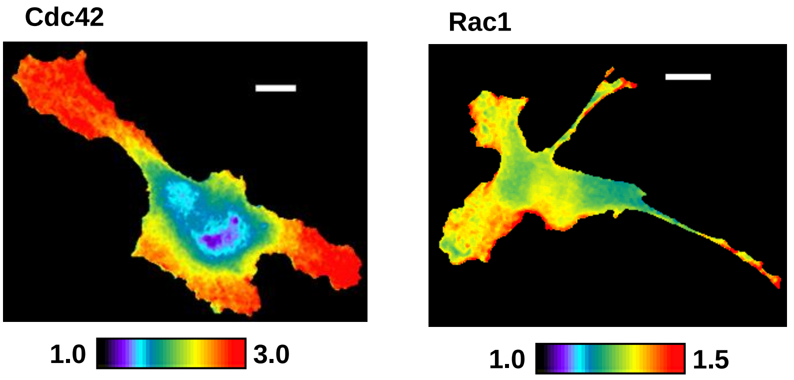

Supplementary Figure 20. Ratiometric images showing spatial distribution of Rac1 and Cdc42 activities as measured using DORA biosensors in migrating MC3T3-E1 cells. Scale bar, 10 μm.

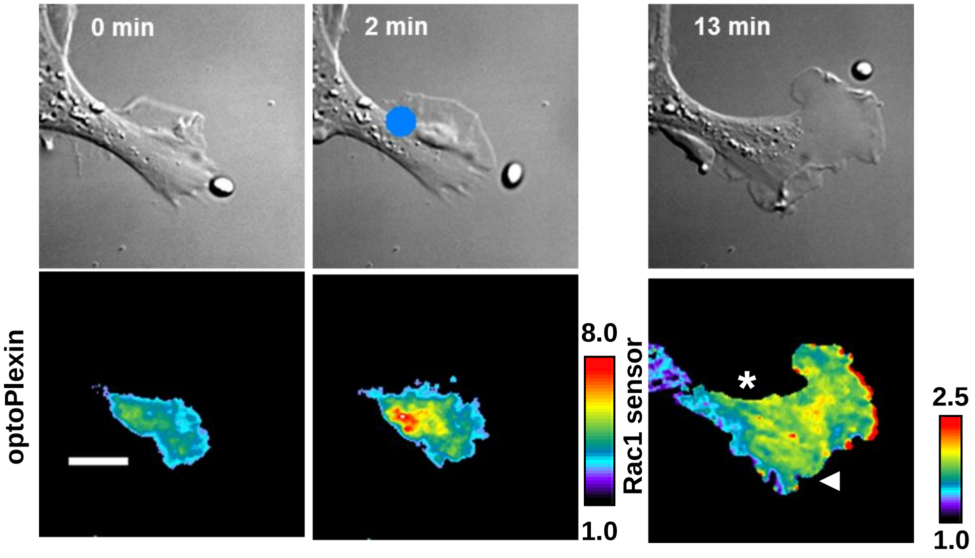

Supplementary Figure 21. DIC and TIRF images showing local activation of mCherry-OptoPlexin and induction of CIL in MC3T3-E1 cells co-expressing Dora-Rac1 biosensor. Upon induction of CIL, TIRF FRET and CFP images were captured and processed to generate ratiometric image indicating Dora-Rac1 sensor activity as displayed in pseudo-colour. Blue circle, region of illumination. Scale bar, 10 μm. Asterisk, induced retraction. Arrowhead, induced protrusion.

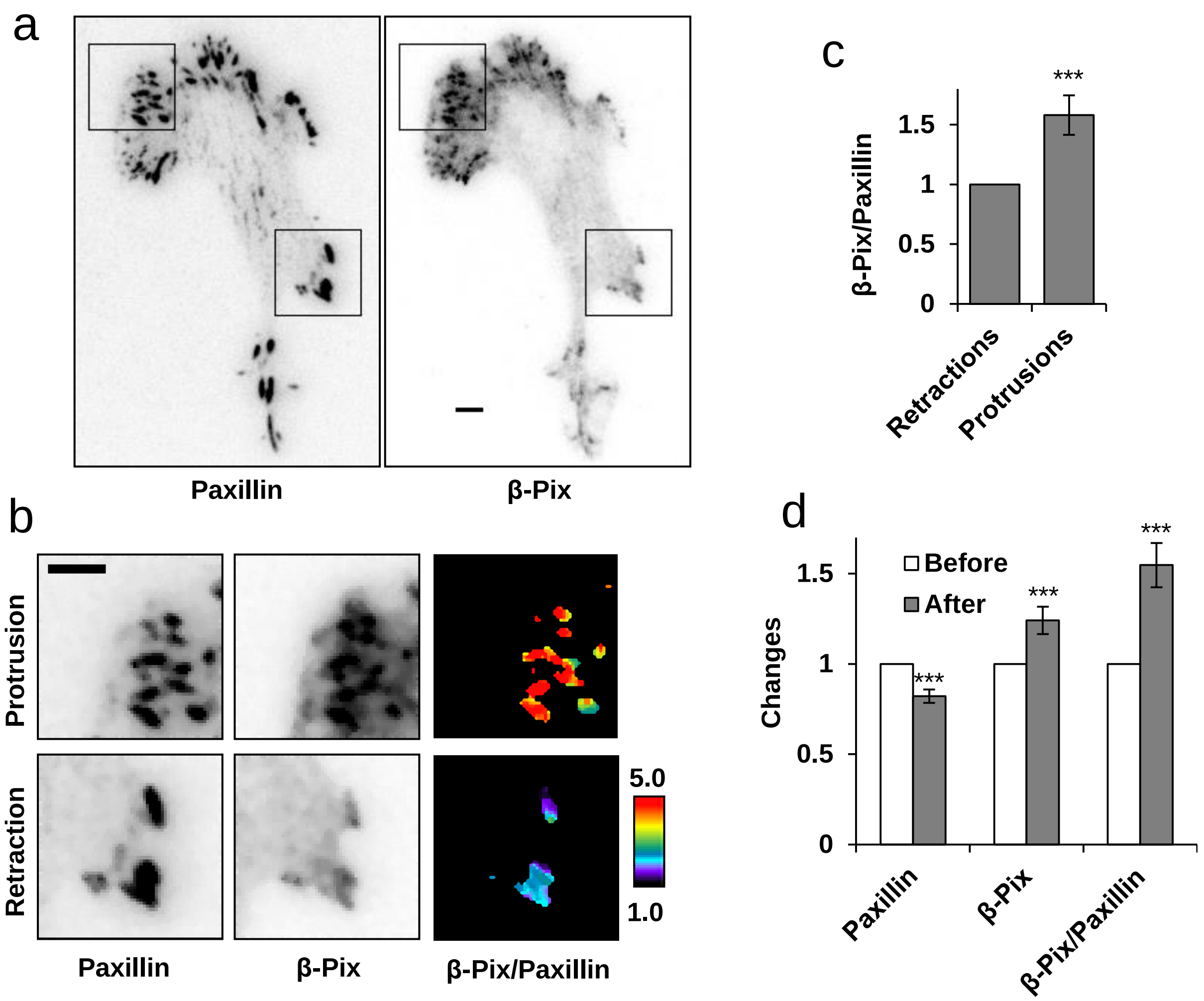

Supplementary Figure 22. (a) Representative TIRF images showing spatial distribution of mCherry-Paxillin and mVenus-β-Pix in a migrating MC3T3-E1 cell. **(b)** Inset squares at a protrusion and a retraction in (a) are magnified, and processed to generate a ratiometric image (mVenus-β-Pix/mCherry-Paxillin), which illustrates the differences in mVenus-β-Pix and mCherry-Paxillin association withnascent and mature adhesions at protrusions and retractions respectively. For (a) and (b) scale bar, 5 μm. **(c)** Normalized ratio of mVenus-β-Pix to mCherry-Paxillin signal in migrating MC3T3-E1 cells at retractions and protrusions. **(d)** Changes in mCherry-Paxillin, mVenus-β-Pix and mVenus-β-Pix/mCherry-Paxillin ratio in MC3T3-E1 cells on 10 μM Y-27632 ROCK inhibitor treatment, normalized to values prior to treatment. n = 7 cells, mean ± s.e.m. *** $p<0.001$, * $p<0.05$, Student's t-test.

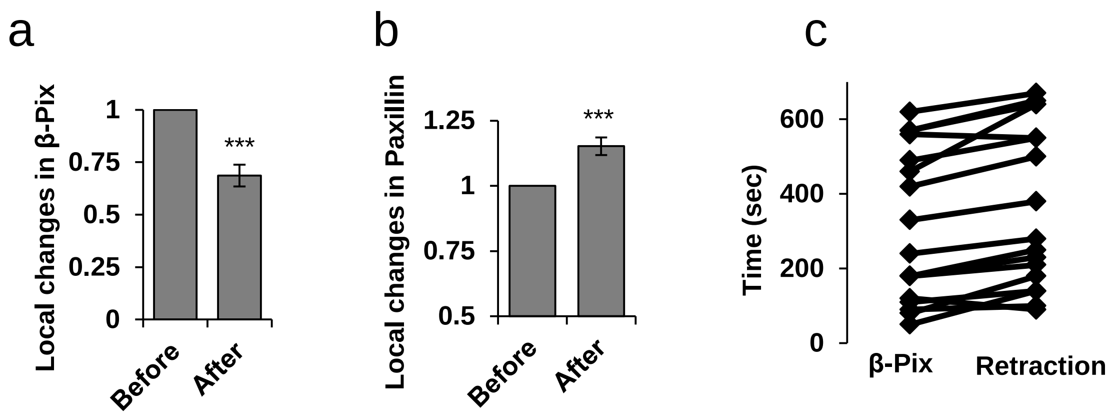

Supplementary Figure 23. (a) Changes in mVenus-β-Pix in a 50 μm diameter circle centered around the region of illumination in MC3T3-E1 cells upon local activation of mCherry-optoPlexin. Mean ± s.e.m, n=18 cells. **(b)** Changes in mCherry-Paxillin in a 50 μm diameter circle centered around the region of illumination in MC3T3-E1 cells upon local activation of mVenus-optoPlexin. Mean ± s.e.m, n=10 cells. **(c)** Delay in initiation of mVenus-β-Pix depletion in a 50 μm diameter circle centered around the region of illumination, and that of retraction upon local activation of mCherry-optoPlexin activation. *** p<0.001, * p<0.05, Student's t-test.

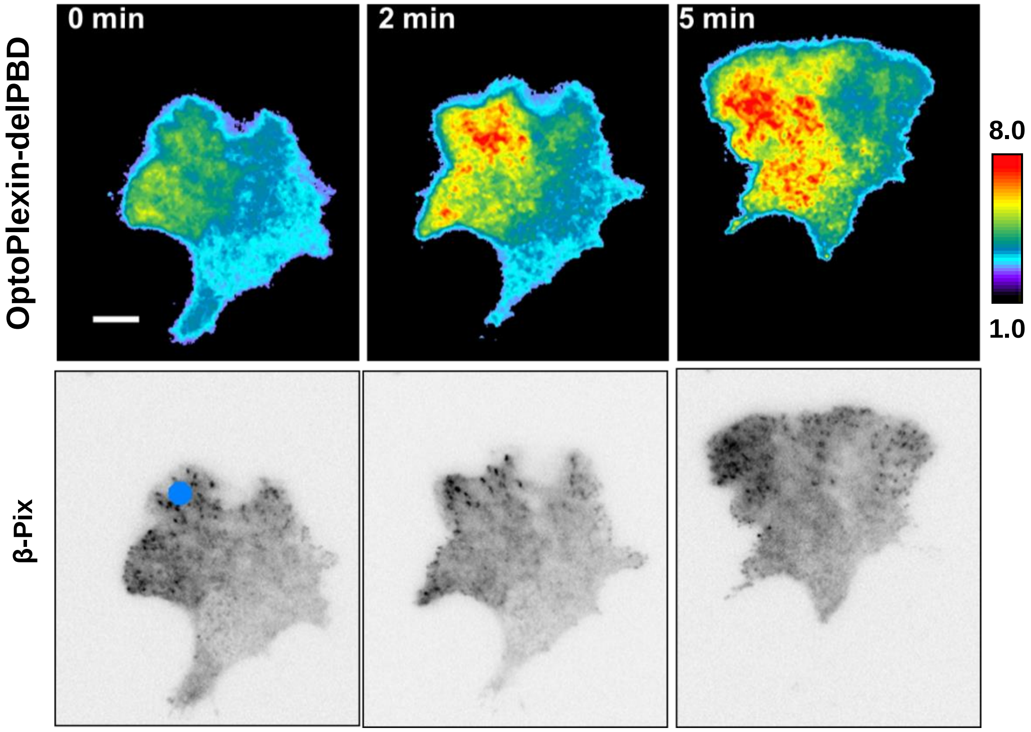

Supplementary Figure 24. Representative TIRF images showing lack of mVenus-β-Pix depletion from the region of localized mCherry-optoPlexin-delPBD activation in MC3T3-E1 cells. Blue circle, region of illumination. Scale bar, 10 μm.

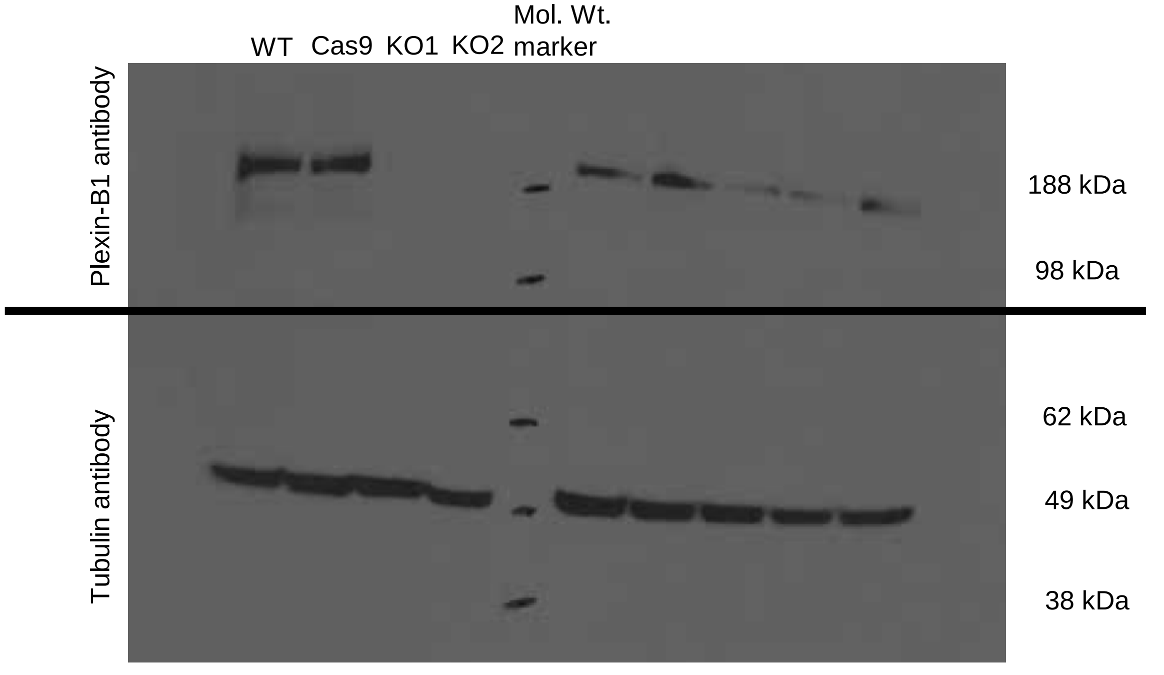

Supplementary Figure 25. Uncropped scan for Figure 1c.

Name	Sequence	Description
sgRNA1 - Fw	CACCGACAGTCCTGTCCCGTAGCAT	sgRNA1 forward primer
sgRNA1 - Re	AAACATGCTACGGGACAGGACTGTC	sgRNA1 reverse primer
sgRNA2 - Fw	CACCGCCGCAGGGTGACGACACACC	sgRNA2 forward primer
sgRNA2 - Re	AAACGGTGTGTCGTCACCCTGCGGC	sgRNA2 reverse primer
PCR Primer 1 - Fw	AAGGAACAAGATTGAGTGTGG	Forward primer upstream of sgRNA1
PCR Primer 2 - Re	CCTGATCTGTCCCAGGCTCCG	Reverse primer downstream of sgRNA2
Sequencing primer	GCCTCTGGGCTCACCAGGAGC	primer (reverse) for sequencing PCR product

Supplementary Table 1. List of primers used in this study for CRISPR-Cas9 mediated Plexin-B1 knock-out
